# Pilomatricoma in Turner Syndrome: Association with Renal Malformations and Analysis of the Vitamin D Pathway

**DOI:** 10.3390/ijms27219485

**Published:** 2026-10-23

**Authors:** Rehotbevely Barrientos, Rodolfo Rodríguez-Jurado, Luz María Torres Espíndola, Marimar Sáez-de-Ocariz, José Velázquez Aragón, Óscar Pérez González, Camilo Villarroel Cortés, Miguel Rodríguez Morales, Sara Frías, Rafael Velázquez Cruz

**Affiliations:** 1 Laboratorio de Citogenética, Instituto Nacional de Pediatría, Mexico City 04530, Mexico; 2 Departamento de Patología, Instituto Nacional de Pediatría, Mexico City 04530, Mexico; 3 Laboratorio de Farmacología, Instituto Nacional de Pediatría, Mexico City 04530, Mexico; 4 Servicio de Dermatología, Instituto Nacional de Pediatría, Mexico City 04530, Mexico; 5 Laboratorio de Oncología Experimental, Instituto Nacional de Pediatría, Mexico City 04530, Mexico; 6 Departamento de Genética Humana, Instituto Nacional de Pediatría, Mexico City 04530, Mexico; 7 Departamento de Medicina Genómica y Toxicología Ambiental, Instituto de Investigaciones Biomédicas, Universidad Nacional Autónoma de México, Mexico City 04510, Mexico; 8 Laboratorio de Genómica del Metabolismo Óseo, Instituto Nacional de Medicina Genómica, Mexico City 14610, Mexico

**Keywords:** pilomatricoma, Turner syndrome, renal malformations, vitamin D, Wnt/β-catenin

## Abstract

Pilomatricoma is a rare benign cutaneous neoplasm that may occur more frequently in syndromic conditions such as Turner syndrome (TS). This study aimed to evaluate the prevalence and clinical characteristics of pilomatricoma and its association with clinical factors, serum vitamin D (VD) levels, and genetic variants in the VD pathway in patients with TS. Clinical variables and serum VD levels were obtained from medical records, and single-nucleotide variants in the *GC*, *NADSYN1*, and *VDR* genes were analyzed by allelic discrimination using real-time polymerase chain reaction. Among 97 patients with TS, six cases of histopathologically confirmed pilomatricoma were identified, corresponding to a prevalence of 6.2%, with lesions predominantly located on the head and upper extremities. Four patients (66.7%) had renal malformations. In exploratory multivariable analysis, renal malformations were significantly associated with pilomatricoma (adjusted OR = 14.2; 95% CI: 2.1–95.4; nominal *p* = 0.008; FDR-adjusted *p* = 0.032). No significant associations were observed with serum VD levels or the analyzed genetic variants. These findings suggest that pilomatricoma may be more frequent in TS than previously reported and identify an exploratory association between pilomatricoma and renal malformations. Given the small number of pilomatricoma cases, this association requires confirmation in larger independent cohorts.

## 1. Introduction

Pilomatricoma or pilomatrixoma is a benign cutaneous neoplasm derived from hair follicle matrix cells, which was first described by Malherbe and Chenantais, and has a low incidence in the general population, where it represents 1–1.59% of all benign skin tumors [1,2]. Pilomatricoma is composed of basaloid cells, which correspond to immature keratinocytes derived from the hair follicle matrix and are characterized by high proliferative activity and the ability to differentiate into keratinized cells [3,4,5,6]. It occurs more frequently in the pediatric population and exhibits a predilection for the head and neck region [5,6,7]. However, its frequency may increase in syndromic conditions, in which it can also present as multiple lesions [8,9]. Turner syndrome (TS) is a prevalent chromosomal disorder characterized by the complete or partial loss of the second sex chromosome and, in addition to short stature and gonadal dysgenesis, is associated with a wide range of clinical manifestations, including skeletal, cardiac, and renal malformations, as well as endocrine–metabolic abnormalities [9,10,11]. In patients with TS, a higher prevalence of pilomatricoma has been reported compared with the general population, reaching up to 2.6% in some case series [7,12]. It is known that vitamin D plays a key role in skin homeostasis by regulating keratinocyte proliferation, differentiation, and apoptosis through its nuclear receptor, the vitamin D receptor (VDR) [13,14]. Furthermore, VDR interacts with the Wnt/β-catenin signaling pathway, which plays a fundamental role in hair follicle regulation and cutaneous tumorigenesis [15]. In this context, based on these biological relationships, genetic variation in vitamin D-related genes may represent a potential source of interindividual variability in vitamin D metabolism and signaling. Therefore, the present study evaluated selected single-nucleotide variants (SNVs) in *GC* (rs7041, rs4588, and rs2282679), *NADSYN1* (rs4944957), and *VDR* (rs10783219 and rs4516035), together with serum 25(OH)D concentrations, in relation to pilomatricoma in patients with TS (Figure 1).

Despite these observations, evidence regarding the relationship among clinical factors, vitamin D levels, genetic variants in the vitamin D pathway, and the development of pilomatricoma in patients with TS remains limited. Therefore, the aim of the present study was to describe the tumor prevalence and clinical characteristics in a cohort of patients with TS, and to evaluate the association of clinical factors and genetic variants in vitamin D pathway genes with the presence of pilomatricoma.

Conceptual representation of vitamin D metabolism and the proposed interaction between VDR and β-catenin in keratinocytes. The upper pathway illustrates vitamin D synthesis and activation, whereas the lower panels represent VDR/β-catenin signaling during follicular homeostasis and a hypothetical state of proliferative Wnt/β-catenin activation. In the present study, serum 25-hydroxyvitamin D [25(OH)D] concentrations and genetic variants in *GC*, *NADSYN1*, and *VDR* were evaluated; however, pathway activation and protein expression in tissue were not directly assessed. Therefore, this figure represents a proposed biological model based on previous evidence rather than a mechanism demonstrated in this cohort.

## 2. Results

Our study population consisted of 97 patients diagnosed with TS, with a mean age at diagnosis of 10 years. Within the cohort, six cases of pilomatricoma were identified, corresponding to a prevalence of 6.2%. The karyotypes identified among these patients included 45,X in three of the cases (50%). The distribution of lesions showed a clear predominance in the cephalic region, with facial involvement observed in 4 of 6 cases (66.7%), primarily affecting the cheek. In two patients, the lesions were located on the upper extremities, including one case with simultaneous involvement of both the face and arm (Table 1).

### 2.1. Histopathological and Clinical–Dermatological Findings in Our Study Population

#### 2.1.1. Histopathological Findings

The diagnosis of pilomatricoma was histopathologically confirmed in all cases. Typical findings included peripheral basaloid cell areas characterized by hyperchromatic nuclei and high cellular density, along with extensive central regions composed of ghost (shadow) cells. Foci of calcification and a foreign body inflammatory reaction were also observed (Figure 2).

#### 2.1.2. Skeletal Abnormalities

All patients presented skeletal abnormalities, including shortening of the fourth and fifth metacarpals, cubitus valgus, micrognathia, broad chest, high-arched palate, hip dysplasia, and scoliosis. The mean number of orthopedic abnormalities was 2.5 per patient (range: 1–4).

#### 2.1.3. Serum Vitamin D Levels

The mean serum vitamin D level was 24.0 ng/mL (range: 19.7–31.7 ng/mL). Five of the six cases (83.3%) had vitamin D levels below 30 ng/mL. According to current guidelines, serum concentrations below 30 ng/mL are considered indicative of vitamin D insufficiency [14].

#### 2.1.4. Malformations in the Six Patients with Pilomatricoma

Three of the six patients (50%) presented with cardiac malformations, while four patients (66.7%) exhibited renal malformations. A detailed description of the defects is summarized in Table 1.

#### 2.1.5. Association Between Clinical Characteristics and Pilomatricoma

Bivariate analysis was performed on the clinical variables, with statistical significance or a trend toward significance observed only for serum vitamin D levels, cardiac malformations, renal malformations, and the number of skeletal abnormalities. No other variables, including karyotype with or without mosaicism and common clinical problems in patients with TS, such as thyroiditis and low bone mineral density, showed statistical significance.

Consequently, the four aforementioned variables were included in a logistic regression model to evaluate their association with pilomatricoma (Table 2).

### 2.2. Association Between SNVs in the GC, NADSYN1, and VDR Genes and Pilomatricoma

No statistically significant associations were identified between the analyzed SNVs and the presence of pilomatricoma in patients with TS; the *GC* variants rs7041 (*p* = 0.62), rs4588 (*p* = 0.75), and rs2282679 (*p* = 0.94) showed no significant associations; similarly, the *NADSYN1* variant *rs4944957* was not associated with pilomatricoma (*p* = 0.70). Regarding the *VDR* gene, the variants rs10783219 (*p* = 0.58) and rs4516035 (*p* = 0.50) also showed no significant associations with pilomatricoma (Table 3).

## 3. Discussion

Pilomatricoma accounts for a small proportion of cutaneous tumors, even in the pediatric population; its frequency increases substantially in syndromic conditions such as TS, where a prevalence of up to 2.6% has been reported [5,12,16]. However, in our study, the prevalence of pilomatricoma was 6.2%, which is more than twice that previously reported in patients with TS. The higher prevalence observed in our cohort may represent a particular characteristic of the study population; however, given the small number of pilomatricoma cases, this finding should be interpreted cautiously. To our knowledge, population-based estimates of pilomatricoma prevalence in Mexico and prevalence estimates according to ethnic origin remain limited. However, differences in clinical presentation between countries have been reported, as described in previous studies [13,16]. In a large retrospective study conducted in Japan, Kinoshita et al. found an average age at presentation that was higher than expected (33.5 years), with a predominance of upper-limb lesions [16]. They suggested that the anatomical distribution of pilomatricoma may vary according to ethnicity and that the predominance of head and neck lesions reported in previous studies could partly reflect the characteristics of the populations evaluated [3,16].

Another reasonable possibility is the underdiagnosis of pilomatricoma in previous studies, particularly before its association with TS was more widely recognized [12]. In this regard, girls with TS at our institute usually receive comprehensive care with regular evaluations, including a detailed physical examination at each visit. Furthermore, given the relatively young age of our cohort, additional cases of pilomatricoma could potentially be identified during longer-term follow-up. Therefore, our findings provide further evidence supporting the association between TS and pilomatricoma and suggest that its frequency may be higher than previously thought; however, the underlying mechanisms responsible for this observation would remain unclear.

In our cohort, we found 15 patients with renal malformations (16.4%), a frequency similar to that reported in other pediatric TS series, ranging from 13.5% to 17.9% [10,17,18,19]. In the multivariable analysis, renal malformations were the only variable significantly associated with the presence of pilomatricoma, with an adjusted OR of 14.2 (95% CI: 2.1–95.4; nominal *p* = 0.008; FDR-adjusted *p* = 0.032). This association was also observed in the initial bivariate analysis, as renal malformations were present in 66.7% of patients with pilomatricoma compared with 12% of those without pilomatricoma (Fisher’s exact test, *p* = 0.0048). This association was unexpected, as, to our knowledge, no direct association between renal malformations and pilomatricoma has previously been reported.

A potential biological hypothesis that could explain this association involves dysregulation of the Wnt/β-catenin signaling pathway, which plays an essential role in embryonic development and tissue homeostasis [4,20]. In the skin, abnormal activation of this pathway contributes to pilomatricoma pathogenesis. In contrast, during nephrogenesis, proteins such as WNT4 and WNT9B regulate key processes, including the mesenchymal-to-epithelial transition, ureteric bud branching, and nephron formation [20,21]. In addition, the interaction between VDR and β-catenin modulates keratinocyte proliferation and differentiation [15]. Taken together, these observations raise the hypothesis that alterations in Wnt/β-catenin signaling could represent a shared biological pathway contributing to both pilomatricoma development and congenital renal abnormalities; however, this mechanism was not experimentally evaluated in the present study and requires further investigation [20,21]. Therefore, the observed association between pilomatricoma and renal malformations should be considered exploratory and requires confirmation in larger, independent cohorts before any potential clinical implications can be established.

On the other hand, the lack of association observed for the remaining variables may be attributable, at least in part, to the low number of events (six pilomatricoma cases), which limited the statistical power to detect significant effects. Furthermore, serum 25(OH)D concentration may not fully reflect local vitamin D biological activity in the skin, which also depends on VDR-mediated signaling and local vitamin D metabolism [15,16]. Although no significant associations were identified for the evaluated variants, genetic variation in vitamin D-related genes can influence vitamin D bioavailability and signaling [11,22]; therefore, the absence of associations in the present study does not exclude a potential contribution of this pathway to pilomatricoma biology. This possibility is biologically plausible given the established interaction between VDR and Wnt/β-catenin signaling in keratinocyte biology and hair follicle homeostasis [15]. It is also possible that other variants within these genes, as well as additional genes involved in vitamin D metabolism and signaling, may contribute to pilomatricoma susceptibility. Therefore, the six SNVs evaluated in the present study represent only a limited portion of the genetic variation within this pathway. These findings do not currently support specific diagnostic, screening, or therapeutic recommendations based on vitamin D status or the evaluated genetic variants. Future mechanistic studies could consider the functional evaluation of vitamin D/VDR and Wnt/β-catenin signaling in relevant cellular or tissue models. In this context, emerging gene-delivery and intracellular-tracking platforms may represent useful methodological options for future functional investigations [23,24].

This study has several limitations, particularly the small number of pilomatricoma cases, the single-center design, and the limited number of genetic variants analyzed. In addition, we did not evaluate VDR expression or Wnt/β-catenin signaling in tumor tissue; therefore, their potential involvement remains hypothetical. To inform the design of a future confirmatory study, a conservative sample-size calculation assuming an OR of 3.0, 80% power, and a two-sided α of 0.05 indicated that approximately 725 patients with TS would be required [25,26]. Given the low frequency of pilomatricoma, such a study would likely require a multicenter design.

## 4. Materials and Methods

### 4.1. Study Population

The study population comprised 97 Mexican patients with cytogenetically confirmed Turner syndrome (TS) who were evaluated at the Human Genetics Department of the National Institute of Pediatrics, Mexico City, Mexico. Clinical and cytogenetic characteristics, serum vitamin D levels, and selected genetic variants related to the vitamin D pathway were evaluated. Pilomatricoma was identified based on compatible clinical findings and confirmed by histopathological examination in all six affected patients. The inclusion criterion was a cytogenetically confirmed diagnosis of TS, regardless of age. Exclusion criteria were refusal to provide written informed consent and/or assent and receipt of a blood transfusion within three months before peripheral blood collection. The study protocol was approved by the Research, Ethics, and Biosafety Committees of the National Institute of Pediatrics (protocol No. 2021/052).

### 4.2. DNA Extraction and Genotyping

Genomic DNA was extracted from peripheral blood samples using the QIAamp^®^ DNA Blood Mini Kit (Qiagen, Hilden, Germany), according to the manufacturer’s instructions. DNA quality and concentration were assessed before molecular analyses. Genotyping of single-nucleotide variants (SNVs) in *GC* (rs7041, rs4588, and rs2282679), *NADSYN1* (rs4944957), and *VDR* (rs10783219 and rs4516035) was performed by real-time polymerase chain reaction using TaqMan^®^ allelic discrimination assays on an Applied Biosystems^®^ real-time PCR system (Applied Biosystems, Foster City, CA, USA). Allele-specific fluorescent probes enabled discrimination of homozygous and heterozygous genotypes. The variants were selected a priori because *GC* encodes the principal vitamin D-binding protein, *NADSYN1* lies within a locus associated with circulating 25-hydroxyvitamin D concentrations, and regulatory variants in *VDR* may influence receptor expression or signaling.

### 4.3. Statistical Analysis

Statistical analyses were performed using SPSS version 29.0. Continuous variables were summarized as means and ranges, whereas categorical variables were expressed as frequencies and percentages. Associations between clinical variables and pilomatricoma were explored using the chi-square test or Fisher’s exact test, as appropriate, and multivariable logistic regression. The multivariable model included serum vitamin D levels, cardiac malformations, renal malformations, and the number of skeletal abnormalities. Because only six pilomatricoma events were available, the multivariable analysis was considered exploratory; adjusted odds ratios (ORs), 95% confidence intervals (CIs), and nominal *p* values were reported to describe effect estimates and their uncertainty.

Associations between SNVs and pilomatricoma were evaluated under a dominant genetic model, comparing carriers of at least one variant allele with the reference homozygous genotype. Genetic association analyses were performed using available genotype data for each SNV; participants with missing genotype data were excluded from the corresponding variant-specific analysis. To account for multiple comparisons, the Benjamini–Hochberg false discovery rate (FDR) procedure was applied separately to two families of hypotheses: four clinical variables and six vitamin D pathway SNVs. An FDR-adjusted *p* value < 0.05 was considered statistically significant. Given the observational design and the limited number of pilomatricoma cases, the associations were interpreted as exploratory and no causal inferences were made.

## 5. Conclusions

In this exploratory single-center cohort, pilomatricoma was identified in 6.2% of patients with Turner syndrome (TS). Renal malformations were associated with the presence of pilomatricoma after FDR correction; however, the estimate was imprecise and was based on only six cases of pilomatricoma. No significant associations were identified between pilomatricoma and serum vitamin D concentrations or the selected variants in *GC*, *NADSYN1*, and *VDR*. Pilomatricoma may represent a clinical finding associated with the presence of renal abnormalities in patients with TS; however, given the small number of cases, this association should be considered exploratory and does not allow causal inference or support the use of pilomatricoma as a clinical marker of renal malformations. These findings require confirmation in larger independent cohorts with broader genetic analyses and molecular evaluation of lesional tissue.

## Figures and Tables

**Figure 1:**
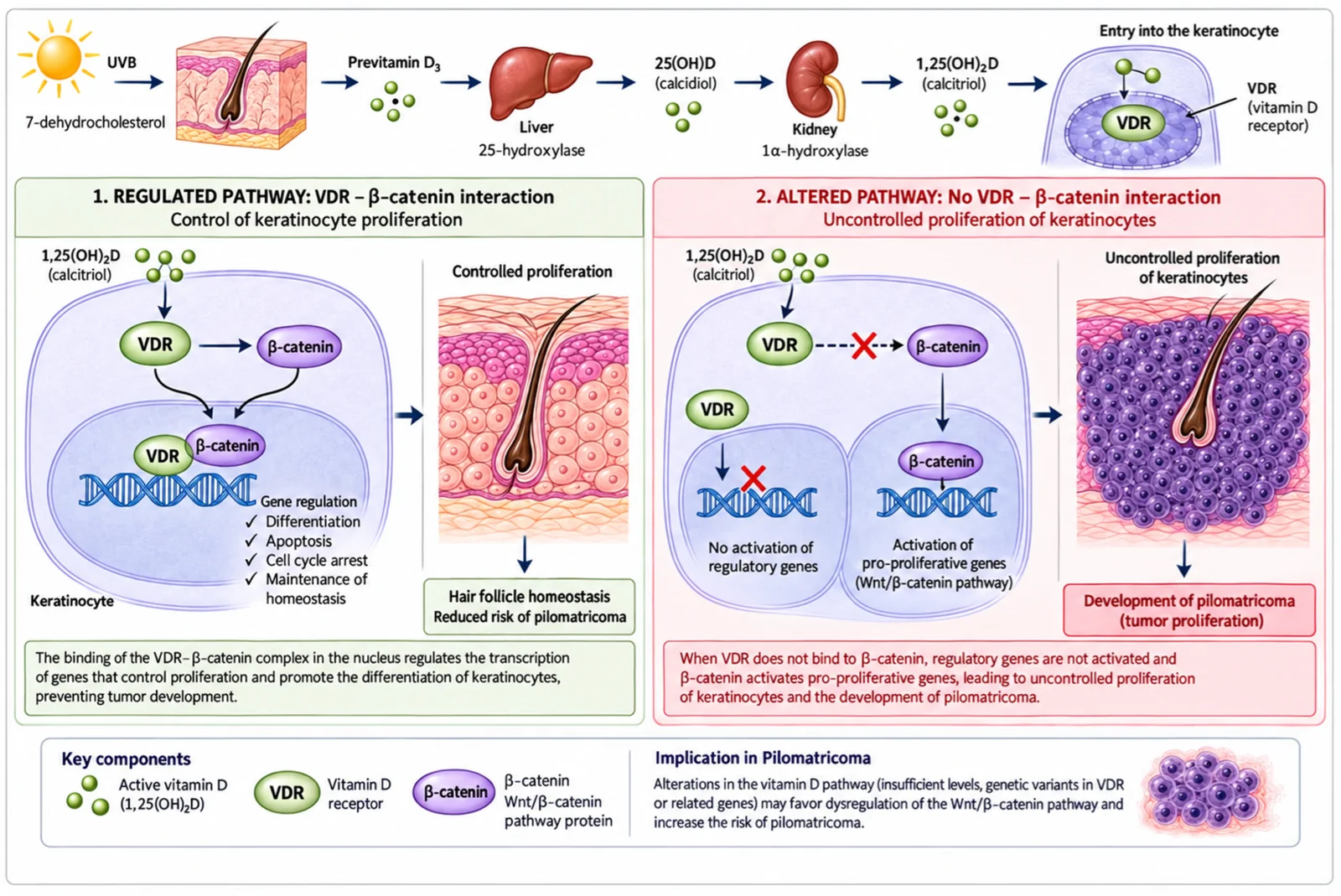
Conceptual framework linking vitamin D/VDR and Wnt/β-catenin signaling to pilomatricoma. Black arrows indicate pathway progression or activation, whereas red crosses indicate loss or inhibition of the corresponding interaction or regulatory step.

**Figure 2:**
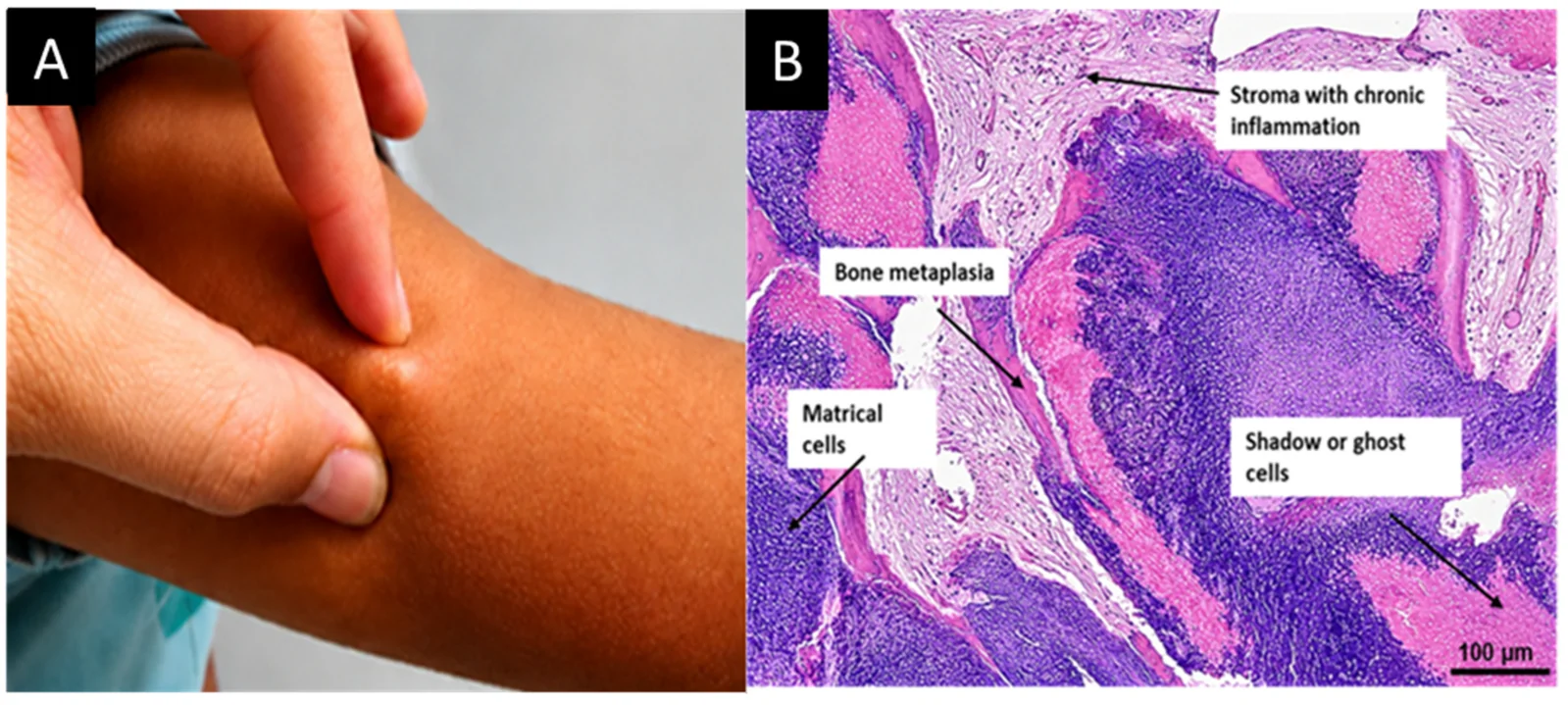
Dermatological and histopathological features of pilomatricoma in a patient with Turner syndrome. (**A**) Subcutaneous nodule on the arm showing the *tent sign* on skin compression. The retrospective clinical photograph was not acquired with a calibrated scale, but it measured 1.3 cm in diameter. (**B**) Hematoxylin and eosin-stained section demonstrating the typical histopathological features of pilomatricoma, including basaloid matrix cells, shadow (ghost) cells, bone metaplasia, and chronic inflammatory stroma. Scale bar = 100 μm.

**Table 1:** Clinical characteristics and vitamin D levels in patients with Turner syndrome and pilomatricoma.

Case	Karyotype	Mean Vit D	CHD	# Skeletal Abn	CRA	Location
1	mos 45,X,i(X)(q10)[5]/45,X[3]/46,XX[42]	19.75	0	3 (SFM, CV, HAP)	0	Right upper arm
2	45,X[40]	23	0	3 (SFM, CV, HAP)	DRCS	Right lower arm
3	45,X[17]	21.65	CoA	2 (SFM, BC)	HK	Cheek and arm
4	46X,i(X)(q10)[20]	23.23	0	1 (HAP)	HK	Cheek
5	mos 45,X(29)/46,XX[64]	24.9	BAV	2 (SFM, HAP)	DRCS	Cheek
6	45,X[115]	31.7	CoA	4 (SFM, CV, BC, HAP)	0	Cheek

Mean Vit D (mean vitamin D level), CHD (congenital heart defects), Skeletal Abn (skeletal abnormalities), CRA (congenital renal anomalies), CoA (coarctation of the aorta), BAV (bicuspid aortic valve), SFM (shortening of the fourth and fifth metacarpals), CV (cubitus valgus), M (micrognathia), BC (broad chest), HAP (high arched palate), DRCS (duplicated renal collecting system), HK (horseshoe kidney).

**Table 2:** Multivariable analysis of clinical characteristics in patients with Turner syndrome with pilomatricoma.

Clinical Characteristic	Adjusted OR	95% CI	*p* (Nominal/FDR)
Serum vitamin D levels	0.94	0.82–1.07	0.34/0.453
Cardiac malformation	1.6	0.3–8.4	0.58/0.580
Renal malformation	14.2	2.1–95.4	0.008/0.032
Number of skeletal abnormalities	1.2	0.8–1.7	0.29/0.453

Renal malformations were significantly associated with the presence of pilomatricoma, with an adjusted OR of 14.2 (95% CI: 2.1–95.4; nominal *p* = 0.008 and FDR-adjusted *p* = 0.032), indicating higher odds of pilomatricoma among patients with renal malformations. In contrast, serum vitamin D levels were not significantly associated with the presence of pilomatricoma (*p* = 0.34). Likewise, no significant associations were observed for cardiac malformations (*p* = 0.58) or the number of skeletal abnormalities (*p* = 0.29) in relation to the presence of pilomatricoma.

**Table 3:** Association of SNVs in genes involved in the vitamin D metabolic pathway with pilomatricoma under a dominant genetic model.

Gene	Variant	Genotype	Cases n (%)	Controls n (%)	OR (95% CI)	*p* (Nominal/FDR)
*GC*	rs7041	A/A + A/C vs. C/C	5 (83.3%)	66 (74.2%)	1.74 (0.18–16.5)	0.62/0.90
*GC*	rs4588	T/T + T/G vs. G/G	5 (83.3%)	43 (48.3%)	1.29 (0.24–6.8)	0.75/0.90
*GC*	rs2282679	T/T + T/G vs. G/G	4 (66.7%)	37 (41.6%)	1.06 (0.20–5.5)	0.94/0.94
*NADSYN1*	rs4944957	G/G + G/A vs. A/A	4 (66.7%)	59 (66.3%)	0.73 (0.14–3.7)	0.70/0.90
*VDR*	rs10783219	A/A+ A/T vs. T/T	3 (50.0%)	46 (51.7%)	0.63 (0.12–3.1)	0.58/0.90
*VDR*	rs4516035	T/T + T/C vs. C/C	3 (50.0%)	49 (55.1%)	0.58 (0.11–2.9)	0.50/0.90

The dominant model compared carriers of at least one variant allele with those homozygous for the reference allele. Percentages were calculated using the number of participants with available genotype data as the denominator; participants with missing genotype data were excluded from the corresponding SNV analysis. OR, odds ratio; CI, confidence interval; FDR, false discovery rate. FDR adjustment was performed across the six SNV comparisons using the Benjamini–Hochberg procedure.

## Data Availability

The raw data supporting the conclusions of this article will be made available by the authors on request.

## Abbreviations

The following abbreviations are used in this manuscript:

TS	Turner syndrome
VD	Vitamin D
VDR	The vitamin D receptor
SNV	Single-nucleotide variant
Mean Vit D	Mean vitamin D
CHD	Congenital heart defects
Skeletal Abn	Skeletal abnormalities
CRA	Congenital renal anomalies
CoA	Coarctation of the aorta
BAV	Bicuspid aortic valve
SFM	Shortening of the fourth and fifth metacarpals
CV	Cubitus valgus
M	Micrognathia
BC	Broad chest
HAP	High-arched palate
DRCS	Duplicated renal collecting system
HK	Horseshoe kidney
